# Antinociceptive and Anti-Inflammatory Activities of the Ethanolic Extract from *Synadenium umbellatum* Pax. (Euphorbiaceae) Leaves and Its Fractions

**DOI:** 10.1155/2013/715650

**Published:** 2013-01-21

**Authors:** Rodrigo Borges, Marcus Vinícius Mariano Nascimento, Adryano Augustto Valladão de Carvalho, Marize Campos Valadares, José Realino de Paula, Elson Alves Costa, Luiz Carlos da Cunha

**Affiliations:** ^1^Faculdade de Farmácia, Universidade Federal de Goiás, Avenida Universitária com 1a Avenida, Quadra 62, 2 Andar, Sala 36, 74605-220 Goiânia, GO, Brazil; ^2^Instituto Nacional de Metrologia, Qualidade e Tecnologia (INMETRO), Avenida Nossa Senhora das Graças, 50 Xerém, Duque de Caxias, 25250-020 Rio de Janeiro, RJ, Brazil; ^3^Instituto de Ciências Biológicas, Universidade Federal de Goiás, Campus II-Samambaia, Saída para Nerópolis (Km 13), Caixa Postal 131, 74001-970 Goiânia, GO, Brazil

## Abstract

*Synadenium umbellatum* Pax., popularly known in Brazil as “cola-nota,” “avelós,” “cancerola,” and “milagrosa”, is a plant species used in folk medicine for the treatment of inflammation, pain, and several diseases. This study aimed to investigate the antinociceptive and anti-inflammatory activities of the ethanolic extract from *Synadenium umbellatum* Pax. leaves (EES) and its hexane (HF), chloroform (CF), and methanol/water (MF) fractions using the acetic acid-induced abdominal writhing test, formalin-induced paw licking test, tail flick test, croton oil-induced ear edema test, and carrageenan-induced peritonitis test. EES and MF reduced the number of acetic acid-induced abdominal writhes, while CF and HF did not. EES effect on acetic acid-induced abdominal writhing was reversed with a pretreatment with naloxone. EES reduced licking time in both phases of the formalin-induced paw licking test, but did not prolong the latency in the tail flick test. These results show that EES presented antinociceptive activity, probably involving the opioid system, anti-inflammatory activity in the croton oil-induced ear edema test, and leukocyte migration into the intraperitoneal cavity. MF also presented anti-inflammatory activity in the croton oil-induced ear edema test. In conclusion, EES and MF have antinociceptive activity involving the opioid system and anti-inflammatory activity.

## 1. Introduction

Medicinal plants are often used in order to replace or assist conventional therapies in the treatment of various diseases. Among other factors, the preference for the use of medicinal plants may be related to their availability and low cost. It is known that medicinal plants have a large diversity of secondary metabolites with different biological activities [1, 2], which justifies the research on pharmacological properties of plant species and their potential uses in drug development.

Despite the preference of pharmaceutical companies for drug development using synthetic routes, in recent decades, a major concern of the market about the therapeutic potential of plants has been observed [3, 4]. This fact has been proven by the evidence that nowadays about 25% of the prescribed drugs in the world are directly or indirectly obtained from plants. In addition, approximately 49% of the drugs developed between 1981 and 2002 were obtained from natural products, or similar semisynthetic or synthetic compounds based on natural products [4].

Brazil is privileged because it ranks first among the richest countries in biodiversity in the world, accounting for 22% of the higher plant species on the planet [5]. The immense variety of plant, animal, and microorganism species in Brazilian ecosystems undoubtedly has important advantages for drug development [6].

Most clinically important medicines are steroidal or nonsteroidal anti-inflammatory drugs for the treatment of inflammatory-related diseases and pain. Although these compounds have potent activity, long-term administration is required to treat chronic diseases. Furthermore, these drugs may have various and severe adverse side effects, such as gastric disorders, kidney, liver, and heart failure, prolonged bleeding after injury or surgery, adrenal suppression, insomnia, redness, increased appetite, Cushing's syndrome, and diabetes. Naturally occurring agents, with high effectiveness and very few side effects, are desirable as an alternative to chemical therapeutic agents [7, 8].

*Synadenium umbellatum* Pax., a member of the family Euphorbiaceae, popularly known as “cola-nota,” “avelós,” “cancerola,” or “milagrosa” in Brazil, has been used in folk medicine as an analgesic, anti-inflammatory, and anticancer agent, among other purposes. The latex of this plant has long been used in traditional Brazilian medicine for the treatment of various different diseases, such as diabetes, Hansen's disease, trypanosomiases, and leukemia [9].

Some species of the genus *Synadenium* are potent inhibitors of prostaglandin synthesis, such as *Synadenium cupularis*, which justifies the use of this plant as an anti-inflammatory and analgesic agent [10]. Several other plants belonging to the family Euphorbiaceae have shown pharmacological activities, such as *Euphorbia kansu*, which has analgesic [11] and antitumor properties [12], and various species of the genus *Phyllanthus*, which have anti-inflammatory and analgesic activities [13]. Moreover, certain species of this family, such as *Bridelia retura* [14], *Alchornea cordifolia* [15], *Euphorbia splendens* [16], among others, present potent anti-inflammatory activity.

The present study aimed to evaluate the antinociceptive and anti-inflammatory activities of the ethanolic extract from *S. umbellatum* leaves, and its hexane, chloroform, and methanol/water fractions, as well as a possible mechanism of action of this ethanolic extract. 

## 2. Materials and Methods

### 2.1. Plant Material

Samples of *S. umbellatum* leaves (17 kg) were collected in Goiânia, GO (16°66′72.03′′S; 49°23′02.09′′W), Brazil, in the summer of 2005/2006. The botanical material was identified and a voucher specimen was deposited in the herbarium of the Universidade Federal de Goiás (no. UFG-27160). The leaves were dried at 40°C in an air circulating drying oven for 48 h and ground in a knife mill.

### 2.2. Extraction

To obtain the ethanolic extract from *S. umbellatum* (EES), the dried leaves were macerated in 95% ethanol (1 : 5 w/v), stirred for 5 h, and filtered. The extraction was repeated twice more. The filtrate was concentrated to dryness under vacuum (at 40°C) with a rotary evaporator (Quimis Q344B), yielding 90 g of EES (6.9%).

### 2.3. Chlorophyll Elimination and Ethanolic Extract Fractionation

This step was carried out according to Ferri [17] with some modifications. An aliquot of EES (45 g) was dissolved in methanol at 4°C, kept at this temperature for 18 h, and filtered. Distilled water at 4°C was added to this solution to a proportion of 7 : 3 (water : methanol, v/v), and the resulting solution was filtered through Celite and partitioned three times with n-hexane (1 : 1) and chloroform (1 : 1), resulting in hexane (HF), chloroform (CF), and methanol/water (MF) fractions. HF and CF were individually concentrated to dryness under vacuum (at 40°C) with a rotary evaporator (Quimis Q344B), yielding 5% and 11.5%, respectively, whereas MF had the methanol evaporated and the final solution was lyophilized, yielding 12.3%. EES, HF, CF, and MF were dissolved in vehicle (2% Tween in saline) prior to administration.

### 2.4. Animals

Adult male albino mice (*Mus musculus*), weighing 25–35 g, obtained from the Chemical Industry of the State of Goiás (IQUEGO), were randomly allocated to treatment groups. The animals were kept under controlled temperature and a 12 h light-dark cycle, with food and water *ad libitum*, and brought to the laboratory at least 1 h before starting the experiments for acclimation to the new environment. Oral and subcutaneous administrations were performed 60 min and 30 min before the pharmacological activity experiments, respectively.

The study was approved by the Local Ethics and Research Committee (CEP-UFG 118/2007). The experiment was conducted in accordance with the Guide for the Care and Use of Laboratory Animals, published by the U.S. National Institute of Health (NIH Publication, revised in 1985) and bioethical principles of the Colégio Brasileiro de Experimentação Animal (COBEA, Brazil) and the Brazilian law (Law no. 11794/2008).

### 2.5. Antinociceptive Tests

#### 2.5.1. Acetic Acid-Induced Abdominal Writhing Test with the Crude Extract

Mice were treated by gavage (*p.o.*) with vehicle (2% Tween in saline; 10 mL/kg), EES (25, 50, or 100 mg/kg), or indomethacin (10 mg/kg). Acetic acid solution (1.2%, v/v; 10 mL/kg) was injected intraperitoneally (i.p.) 60 min after the groups were treated. The number of writhes—a response consisting of contraction of the abdominal wall and pelvic rotation followed by hind limb extension—produced in each animal by the acetic acid injection was counted for 30 min immediately after the acetic acid administration [18].

#### 2.5.2. Influence of Pretreatment with Naloxone

The positive control was fentanyl (100 mg/kg) and the negative control was the vehicle (2% Tween in saline; 10 mL/kg). Animals were pretreated with naloxone (3 mg/kg, s.c.), a nonselective opioid antagonist, 15 min before being treated with the negative control or EES (100 mg/kg only).

#### 2.5.3. Formalin-Induced Paw Licking Test with the Crude Extract

The experimental groups of mice were treated by gavage (*p.o.*) with vehicle (2% Tween in saline; 10 mL/kg), EES (100 mg/kg), or indomethacin (10 mg/kg) 60 min before the administration of 20 *μ*L formalin (3% v/v) in the right hind paw, or subcutaneously (s.c.) with morphine (10 mg/kg) 30 min before the treatment. After the phlogistic agent injection, the mice were individually placed into a transparent acrylic box and a mirror was positioned under the box to enable unhindered observation of the formalin-injected paw for 30 min. The pain reaction time (licking time) was assessed in two periods: 0 to 5 min—the first phase (neurogenic pain caused by direct stimulation of the nociceptors), and 15 to 30 min—the second phase (inflammatory pain caused by release of inflammatory mediators) [19].

#### 2.5.4. Tail Flick Test with the Crude Extract

This assay allows the study of drugs with spinal analgesic activity by evaluating the time, in seconds (s), that the animal takes to remove the tail of the local impact of a painful thermal stimulus. This noxious stimulation was produced by immersion of the terminal 3 cm of each mouse's tail in water bath at 55.5 ± 0.5°C [20]. Groups had been previously selected for their reactivity to noxious stimulation. Mice whose response time was longer than 7 s were not used. The reactivity was measured every 30 min, beginning 60 min before, and continuing for 2 h after the administration of vehicle (2% Tween in saline; 10 mL/kg, *p.o.*), EES (25, 50, or 100 mg/kg, *p.o.*), or morphine (10 mg/kg, s.c.). To avoid injuries, the maximum time the tails remained in contact with the thermal nociceptive stimulus was 20 s (cut-off).

#### 2.5.5. Acetic Acid-Induced Abdominal Writhing Test with the Fractions

Mice were treated by gavage (*p.o.*) with vehicle (2% Tween in saline; 10 mL/kg), HF (10 mg/kg), CF (20 mg/kg), or MF (25 mg/kg) 60 min before i.p. administration of acetic acid solution (1.2%, v/v; 10 mL/kg,). The number of writhes provoked in each animal by the acetic acid injection was counted for 30 min immediately after the administration [18]. Fraction yield ratio after purification from the crude extract was about 1 : 2 : 2.5 for HF, CF, and MF, respectively. Therefore, dose fractions used in the present experiments reflect the same proportionality, that is, 10, 20, and 25 mg/kg of HF, CF, and MF, respectively.

### 2.6. Anti-Inflammatory Tests

#### 2.6.1. Carrageenan-Induced Peritonitis Test with the Crude Extract

This test assesses the leukocyte migration into the peritoneal cavity and several other parameters [21]. Experimental groups were treated orally with vehicle (2% Tween in saline; 10 mL/kg), EES (25, 50, or 100 mg/kg), or dexamethasone (2 mg/kg). Aliquots of 0.25 mL of carrageenan (1% w/v in saline) were injected i.p. 60 min after the treatment. The animals were euthanized 4 h after carrageenan administration and 2 mL of modified phosphate buffered saline (PBS with heparin, 10 IU/mL) were injected into their peritoneal cavity. Total cell counts in the peritoneal lavage fluid were performed using a Neubauer counting chamber.

#### 2.6.2. Croton Oil-Induced Ear Edema Test with the Crude Extract and MF Fraction

Mice were orally treated with vehicle (2% Tween in saline; 10 mL/kg), EES (25, 50, or 100 mg/kg), MF (6, 12, or 25 mg/kg), or dexamethasone (2 mg/kg). Aliquots of 20 *μ*L of croton oil (2.5% v/v in acetone solution) were topically applied to the right ear of each mouse 60 min after the treatment. The same volume of acetone was also topically applied to their left ear. After 4 h, the animals were euthanized by cervical dislocation and a 6 mm diameter disk was removed from each ear and weighed on an analytical scale [22]. The results of the weight difference between the disks of both ears of each animal were compared with the experimental control group.

### 2.7. Statistical Analysis

Data are expressed as mean ± SEM. Statistical analysis was performed using ANOVA followed by Dunnett's test. *P* values less than 0.05 (*P* < 0.05) were considered significant. The data obtained were analyzed using the GraphPad software program Version 4.0.

## 3. Results 

### 3.1. Acetic Acid-Induced Abdominal Writhing Test with the Crude Extract

**Figure 1:**
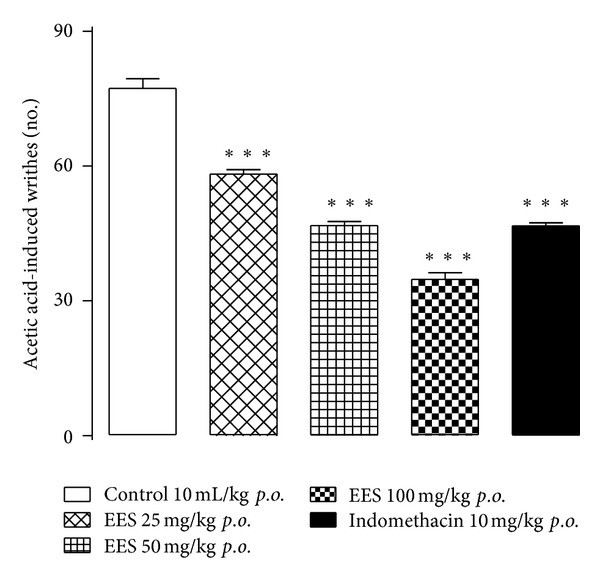
Analgesic effect of the ethanolic extract from *Synadenium umbellatum* leaves (EES; 25, 50, and 100 mg/kg) on the number of acetic acid-induced abdominal writhes in mice (*n* = 6). Vertical bars represent means ± SEM. Indomethacin (10 mg/kg, *p.o.*) was used as positive control. ***Statistically different from the control group (*P* < 0.001)—ANOVA and Dunnett's test.

EES showed significant reduction in the number of abdominal writhes in mice induced by acetic acid administration in a dose-response manner compared to the control group. Pretreatment with EES at the doses of 25, 50, or 100 mg/kg reduced the number of abdominal writhes by 24.7%, 39.5%, and 55.0%, respectively, and the pretreatment with indomethacin (10 mg/kg) reduced this number by 39.6% (Figure 1).

### 3.2. Influence of Pretreatment with Naloxone

**Figure 2:**
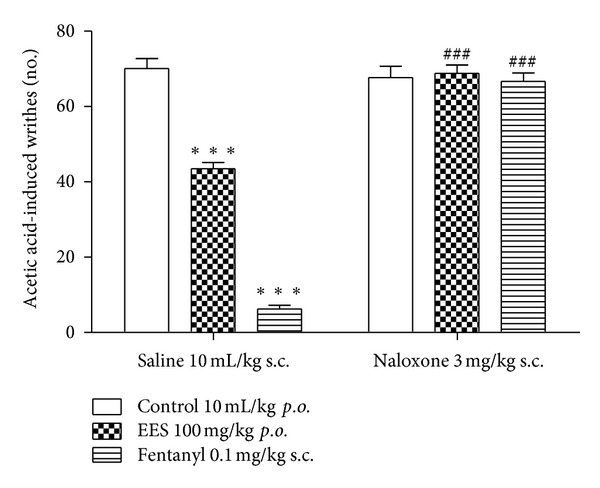
Effect of pretreatment with naloxone on the analgesic effect of the ethanolic extract from *Synadenium umbellatum* leaves (EES; 100 mg/kg) to reduce the number of acetic acid-induced abdominal writhes in mice (*n* = 6). Vertical bars represent means ± SEM. ***Statistically different from the control group (*P* < 0.001)—ANOVA and Dunnett's test. ^###^Statistically different from the treated group (*P* < 0.001)—ANOVA and Dunnett's test.

Preadministration of the opioid antagonist naloxone (3 mg/kg, s.c.) to the group treated with saline (*p.o.*) did not change the number of abdominal writhes induced by acetic acid 30 min after administration compared to the group pretreated with saline. The treatment with the opioid agonist fentanyl (0.1 mg/kg, s.c.) significantly reduced the number of abdominal writhes induced by acetic acid. However, the administration of naloxone 15 min before fentanyl injection blocked the antinociceptive effect of the latter to control acetic acid-induced abdominal writhing. A similar result was evidenced in the group treated with EES at the dose of 100 mg/kg (*p.o.*) since the pretreatment with naloxone significantly inhibited its analgesic effect (Figure 2).

### 3.3. Formalin-Induced Paw Licking Test with the Crude Extract

**Figure 3:**
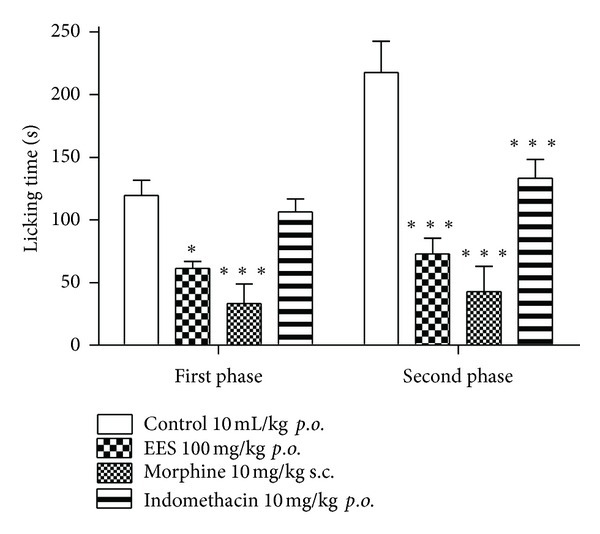
Effects of the ethanolic extract from *Synadenium umbellatum* leaves on the results of the formalin-induced paw licking test in mice (*n* = 6). First phase: 0–5 min; second phase: 15–30 min. Vertical bars represent mean ± S.E.M. *Statistically different from the control group (*P* < 0.05)—ANOVA and Dunnett's test. ***Statistically different from the control group (*P* < 0.001)—ANOVA and Dunnett's test.

Intraplantar injection of 3% formalin into mice hind paw produced intense nociception in two distinct phases. Pretreatment with EES (100 mg/kg, *p.o.*) significantly reduced the first and second phase pain by 48.7% and 73.1%, respectively. In the group treated with morphine, both phases were also significantly reduced, the first by 72.1% and the second by 84.0%. The group treated with indomethacin (10 mg/kg, *p.o.*) did not have the first phase reduced, but the second was significantly reduced by 50.9% (Figure 3).

### 3.4. Tail Flick Test with the Crude Extract

Pretreatment with EES (25, 50, or 100 mg/kg) did not modify the reactivity of the animals to painful thermal stimulation within 2 h of administration (data not shown). Under similar conditions, treatment with morphine significantly increased latency to thermal stimulation 30 min after administration and the antinociceptive effect was maintained during the entire period of evaluation (data not shown).

### 3.5. Acetic Acid-Induced Abdominal Writhing Test with the Fractions

**Figure 4:**
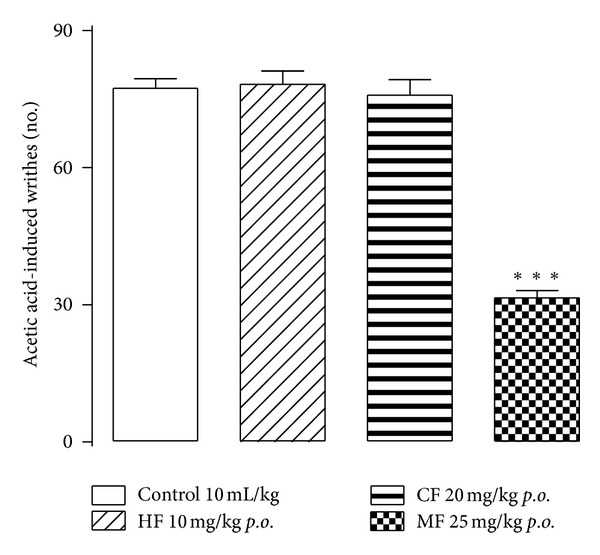
Analgesic effect of the fractions of the ethanolic extract from *Synadenium umbellatum* leaves (HF: hexane fraction; CF: chloroform fraction; MF: methanol/water fraction) on the number of acetic acid-induced abdominal writhes in mice (*n* = 6). Vertical bars represent means ± SEM. ***Statistically different from the control group (*P* < 0.001)—ANOVA and Dunnett's test.

Pretreatment with HF (10 mg/kg) and CF (20 mg/kg) did not alter the number of writhes, but the use of MF (25 mg/kg) significantly reduced this parameter by 59.2% (Figure 4).

### 3.6. Carrageenan-Induced Peritonitis Test with the Crude Extract

**Figure 5:**
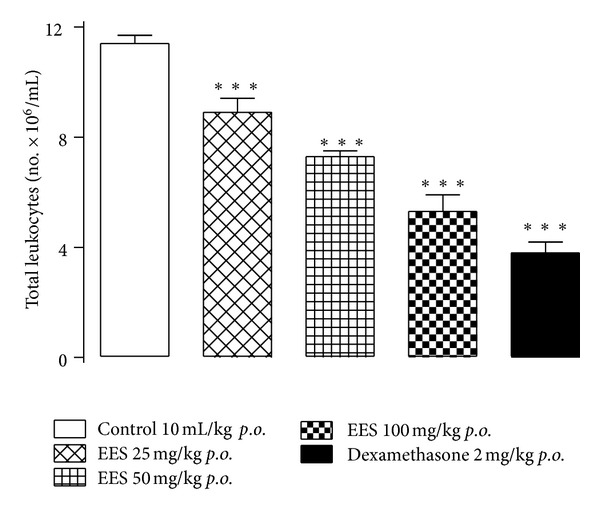
Effect of the ethanolic extract from *Synadenium umbellatum* leaves on leukocyte migration using the carrageenan-induced peritonitis test in mice (*n* = 8). Number of total leukocytes per mL (×10^6^) of peritoneal lavage fluid after carrageenan injection in mice previously treated with: vehicle, EES (25, 50, or 100 mg/kg), or dexamethasone (2 mg/kg). Vertical bars represent means ± SEM. ***Statistically different from the control group (*P* < 0.001)—ANOVA and Dunnett's test.

Using the carrageenan-induced peritonitis test, EES showed significant inhibition of total leukocyte migration in a dose-dependent manner. Pretreatment with EES (25, 50, or 100 mg/kg) reduced leukocyte migration by 21.9%, 36.0%, and 53.5%, respectively, and pretreatment with dexamethasone reduced it by 66.7% (Figure 5).

### 3.7. Croton Oil-Induced Ear Edema Test with the Crude Extract and MF Fraction

**Figure 6:**
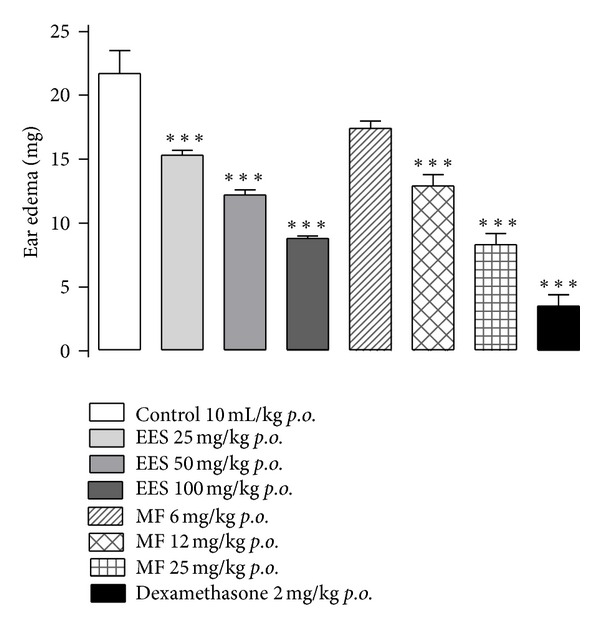
Effect of the ethanolic extract from *Synadenium umbellatum* leaves (EES 25, 50, or 100 mg/kg) and its methanolic/water fraction (MF; 6, 12, or 25 mg/kg) on croton oil-induced ear edema in mice (*n* = 7). Vertical bars represent means ± SEM. ***Statistically different from the control group (*P* < 0.001)—ANOVA and Dunnett's test.

EES and MF significantly reduced the croton oil-induced ear edema in a dose-dependent manner. Pretreatment with EES (25, 50, or 100 mg/kg, *p.o.*) reduced the edema by 30.0%, 43.8%, and 59.4%, respectively. Pretreatment with MF (6, 12, or 25 mg/kg, *p.o.*) also reduced the edema by 19.8%, 41.0%, and 61.9%, respectively. Pretreatment with dexamethasone reduced the edema by 83.9% compared to the control group (Figure 6).

## 4. Discussion

When tissues and cells receive any harmful stimulation, protons (H+), prostaglandin E2 (PGE_2_), serotonine (5-HT), among others, may be released and consequently cause local pain. Acetic acid itself may cause pain and simultaneously it can also stimulate the tissue to produce PGE_2_, thereby causing more pain [23]. Thus, the acetic acid-induced abdominal writhing test is a suitable method widely used to screen and study the antinociceptive activity of different compounds [24]. Although this test is relatively simple and has little specificity, it allows easy observation, it is fast, and presents great sensitivity to various analgesic, nonsteroidal, and steroidal anti-inflammatory drugs, as well as morphine-like compounds and other analgesic substances that act centrally or peripherally.

Furthermore, the results obtained in tests with various classes of analgesic drugs using this model show good correlation with the analgesic action found in other preclinical models and in clinical studies [25–30]. Our results show that EES (25, 50, and 100 mg/kg) reduced the number of acetic acid-induced abdominal writhes. However, because the acetic acid-induced abdominal writhing test has low specificity for antinociceptive responses, additional tests are required to interpret the results obtained, since a wide range of primarily nonanalgesic compounds, such as antihistamines, parasympathomimetic drugs, central nervous system stimulants, monoamine oxidase inhibitors, serotonin antagonists, muscle relaxants, and neuroleptics may also inhibit writhing [31–35].

Due to the need of additional tests to overcome the low specificity of the acetic acid-induced abdominal writhing test, and also aiming to better characterize the antinociceptive activity of the EES, we used the formalin-induced paw licking test in mice, a chemical model of nociception which provides a more specific response [36, 37] and is considered the closest model for clinical pain [38]. The application of the irritant compound into the hind paw makes the nociceptive response more specific, since during grooming the animals most frequently use their forelegs [37]. Following the recommendations of Hunskaar et al. [39] and Murray et al. [40], only the licking was counted in our experiments.

The formalin-induced paw licking test demonstrates two distinct phases of nociceptive behavior which seem to involve different mediators [39, 41–45]. The first phase of nociception starts immediately after formalin injection, extending for the following 5 min, and is believed to occur due to direct chemical stimulation of nociceptors [45], predominantly afferent C fibers and partly, A*δ* fibers [46]. This first phase is inhibited by opioid agonists, such as morphine and fentanyl, by bradykinin B_1_ and B_2_ receptor antagonists, by N-methyl-D-aspartic-acid (NMDA) receptors, as well as by vanilloid receptor antagonists [36, 41, 43, 47, 48]. The second phase of this model takes place 15 to 30 min after formalin injection and is related to the release of several proinflammatory mediators [41].

Since our objectives in using the formalin-induced paw licking test were just to answer whether the reduced number of writhes induced by acetic acid was due to antinociceptive activity and to characterize the type of antinociceptive response, we chose the higher dose of EES (100 mg/kg) to perform the test. We observed that EES (100 mg/kg) strongly inhibited both the first and the second phase of the formalin model, and therefore confirmed that *S. umbellatum *is endowed with potent antinociceptive and/or anti-inflammatory activity.

The acetic acid-induced abdominal writhing test was also used to study the antinociceptive activity mechanism of EES. Based on this approach, we found that the pretreatment with naloxone, a nonselective opioid antagonist able to antagonize opioid receptors located both in the central and peripheral nervous system, inhibited EES antinociceptive activity. These results indicate that the opioid system (central, peripheral, or both) is involved in EES antinociceptive activity.

Aiming to study the spinal antinociceptive action, we performed the tail flick test. This model, similarly to the hot plate test [20, 49], measures animal nociceptive response latencies to thermal stimulus. The model is sensitive to opioid-like drugs and the analgesic activity is mediated by *μ*, *κ*, and *δ* receptors, located in the central nervous system only, but not by the receptors located in the peripheral nervous system [50–52]. Treating the animals with EES, at the same doses used in the acetic acid-induced abdominal writhing test, did not alter mouse latency to painful thermal stimulus. Therefore, we can exclude a spinal opioid system-dependent mechanism (opioid receptors located in the central nervous system). Taking into consideration that the involvement of EES action in the opioid system (centrally or peripherally located) was confirmed after naloxone administration followed by the acetic acid-induced abdominal writhing test and that the tail flick test proved lack of involvement of the opioid system located centrally, it is possible to affirm that the opioid-like effect of EES involves the peripheral opioid system. Given that drugs acting in peripheral opioid system have minimum access to the central nervous system, they can be very important in clinical practice because the typical adverse effects of opioids on the central nervous system (respiratory depression and addiction) are abolished or reduced.

According to our results, MF was the only fraction able to inhibit acetic acid-induced writhing, proving to be responsible for the antinociceptive and/or anti-inflammatory effects observed in EES.

Aiming to examine EES anti-inflammatory activity, we used the carrageenan-induced peritonitis test to assess the migration of total leukocytes to the site of inflammation. Carrageenan, a sulfated polysaccharide, triggers acute inflammation involving the sequential release of various pro-inflammatory mediators, especially histamine, serotonin, kinins, prostaglandins, and thromboxanes [53, 54]. Leukocyte migration is mainly related to the action of leukotrienes, particularly leukotriene-B4, released by lipoxygenase activity, especially in leukocytes [55].

This model of acute inflammation allows the quantification of leukocytes that migrate into the peritoneal cavity under the action of chemotactic agents, mainly leukotrienes and interleukins, and is sensitive to the action of steroidal anti-inflammatory drugs [56–59].

Oral administration of EES at the same doses used in the acetic acid-induced abdominal writhing model reduced the number of leukocytes that migrated into the peritoneal cavity in a dose-dependent manner, following the profile of dexamethasone, used as positive control, suggesting that EES exhibits anti-inflammatory activity.

To check the antiedematous activity of EES and to test MF activity, the croton oil-induced ear edema test was chosen. MF was evaluated for anti-inflammatory activity since it was the only fraction capable of significantly reducing the number of abdominal writhes induced by acetic acid. In this test, the intensity of the edema induced by the topical application of an irritant compound to the outer surface of the ear is evaluated as a parameter of anti-inflammatory activity of the substances administered prior to it. Croton oil is an irritant substance that promotes leukocyte migration causing edema [60], and has tetradecanoylphorbol acetate in its composition, one of the compounds responsible for its irritant action and edema formation, also related to increased vascular permeability and plasma exudation [61, 62].

Examining the results obtained in the croton oil-induced edema test, it may be noted that the pretreatment of mice with EES and MF, at different doses, as well as with dexamethasone, the positive control, reduced edema formation in a dose-dependent fashion. This anti-inflammatory effect can reduce leukocyte migration to inflammatory foci, suggesting the possible interference of EES in chemotactic mechanisms.

## 5. Conclusion

In conclusion, our results show that EES exhibits antinociceptive and anti-inflammatory activities. The antinociceptive activity of EES involves the action of the opioid system, whereas the anti-inflammatory activity involves mechanisms that are able to reduce the formation of edema and the number of leukocytes that migrate to inflammatory foci. MF is the fraction responsible for EES antinociceptive and anti-inflammatory activities. These results may justify the use of *Synadenium umbellatum* Pax. (Euphorbiacea) in folk medicine as an analgesic and anti-inflammatory agent.
